# Factors associated with gastrointestinal obstruction in phytobezoar patients: an emergency ultrasound study

**DOI:** 10.3389/fmed.2026.1843617

**Published:** 2026-06-26

**Authors:** Qingyun Dong, Haxia Qi, Yitong Chai, Yanfen Chai

**Affiliations:** Department of Emergency, Tianjin Medical University General Hospital, Tianjin, China

**Keywords:** associated factors, emergency ultrasound, gastrointestinal obstruction, phytobezoar, retrospective study

## Abstract

**Introduction:**

Phytobezoars are a common cause of acute abdominal pain in northern China and may lead to serious complications including gastrointestinal obstruction (GIO). Early identification of factors associated with GIO is essential for timely intervention. This study aimed to investigate the ultrasound characteristics of phytobezoars and identify factors associated with GIO.

**Methods:**

A retrospective analysis was conducted on 162 patients with ultrasound-diagnosed phytobezoars in the emergency department, divided into GIO (n = 36) and non-GIO (n = 126) groups. Demographics, bezoar characteristics, and ultrasound findings were compared. Univariate analyses (Chi-square test, independent t-test, and Mann-Whitney U-test) and binary logistic regression were used to identify factors associated with GIO. Firth penalized logistic regression including location and a subgroup analysis restricted to pyloric/small-intestinal bezoars were performed as sensitivity analyses.

**Results:**

Phytobezoars occurred predominantly in elderly females, with peak incidence from October to April. Compared with the non-GIO group, the GIO group had a higher proportion of males (*p* < 0.05), higher median age (68 vs. 63 years, *p* < 0.05), and smaller bezoar diameter (41.11 ± 8.57 vs. 48.20 ± 13.75 mm, *p* = 0.004). Notably, all GIO cases had bezoars located in the pylorus or small intestine, whereas none of the 101 gastric body bezoars were associated with obstruction. The primary logistic regression model (excluding location due to complete separation) identified male sex (OR = 3.761), age (OR = 1.054), and bezoar diameter (OR = 0.961) as associated factors (all *p* < 0.05). However, after accounting for location-the dominant predictor of GIO (OR = 220.30, *p* < 0.001)-these associations were no longer statistically significant.

**Discussion:**

The incidence of phytobezoars follows distinct seasonal patterns. Bezoar location is the strongest determinant of GIO, and the associations of sex, age, and diameter observed in the primary model appear to be partly confounded by location. Emergency ultrasound enables rapid identification of bezoar location at the bedside, which may assist in early clinical decision-making. Prospective studies with independent diagnostic confirmation are needed to validate these findings.

## 1 Introduction

Phytobezoars, a specific type of gastric bezoar, frequently contribute to acute abdominal pain in northern China during the winter and spring months. This condition is associated with the consumption of foods such as hawthorn, persimmons, and black jujubes, which are high in tannic acid. Tannic acid interacts with stomach acid and proteins to form insoluble

tannic acid-protein complexes. These complexes precipitate in the stomach, accumulating with food residues to form solid lumps (1). Phytobezoars are the most prevalent type of bezoar and account for the majority of reported cases, with a reported incidence varying considerably across ethnic groups and geographic regions depending on dietary habits (2, 3). Gastric bezoars can lead to a series of gastrointestinal symptoms, ranging from mild abdominal pain and vomiting to severe gastrointestinal complications such as gastrointestinal obstruction, bleeding, or even perforation (4). Bezoars are responsible for 0.4%−4% of cases of mechanical intestinal obstruction (5). Small bowel obstruction is a common surgical condition with diverse etiologies, among which bezoars represent an uncommon but clinically important cause (6–8), frequently necessitating endoscopic or surgical intervention (3, 9). Consequently, the prevention of severe complications arising from phytobezoars hinges on early diagnosis, appropriate treatment, and the identification and management of risk factors to facilitate timely intervention.

Ultrasound, as an essential non-invasive and convenient diagnostic tool in the emergency room, is the preferred imaging means for patients presenting with abdominal pain. Ultrasound imaging effectively displays gastric volume, the precise location of bezoars, their quantity and diameter, as well as the presence of gastric wall injuries and complications such as gastrointestinal obstruction. Additionally, it may serve as a non-invasive tool for follow-up assessment, although dynamic monitoring was not evaluated in the present study. Sonography has been shown to reveal bezoars in approximately 88% of patients, demonstrating characteristic features including an intraluminal hyperechoic arc-like surface with posterior acoustic shadowing (10, 11). Although CT is considered more accurate for confirming the diagnosis and detecting multiple bezoars (12), ultrasound remains particularly valuable in the emergency setting as a rapid, non-invasive screening tool (10).

Most existing studies on bezoars have focused on retrospective analyses from a clinical diagnostic standpoint, emphasizing treatment strategies and outcomes (3, 13, 14), with limited focus on the ultrasound characteristics of bezoars and their association with clinical outcomes. Therefore, the authors conducted a review of data pertaining to emergency ultrasound diagnoses of bezoars at their institution, using the incidence of gastrointestinal obstruction as an outcome measure, to investigate factors associated with gastrointestinal obstruction. This investigation aims to clarify the clinical relevance of bezoar location and other ultrasound characteristics in the emergency setting.

## 2 Methods

In this study, clinical and ultrasound data from 162 patients with phytobezoars, collected between January 2022 and March 2025 at a single tertiary hospital, were retrospectively analyzed. Eligible cases were identified by searching the emergency ultrasound database using the keyword “bezoar.” For patients with multiple emergency visits for phytobezoars during the study period, only the initial presentation was included to avoid duplicate enrollment. All cases were ascertained by emergency ultrasound rather than by an independent reference standard. Data collected included age at onset, sex, year and month of onset, as well as the number, maximum diameter, and location of bezoars.

Inclusion criteria were as follows: (1) patients presenting with abdominal pain to the emergency department between January 2022 and March 2025; (2) ultrasound-based diagnosis of phytobezoar based on the sonographic criteria described below; (3) a self-reported history of recent consumption of hawthorn, persimmons, or black jujubes, as documented by the attending emergency physician during the initial clinical assessment; and (4) sufficient clinical and ultrasound data to determine case ascertainment, bezoar location, and GIO status. Cases with missing values for a specific covariate were excluded only from analyses requiring that covariate. Exclusion criteria were: (1) patients with other types of bezoars (trichobezoar, lactobezoar, or pharmacobezoar); (2) patients with incomplete ultrasound data; and (3) patients with coexisting gastrointestinal malignancies; and (4) recurrent presentations of the same patient during the study period.

Diagnostic criteria for phytobezoar on ultrasound included: an intraluminal hyperechoic mass with strong posterior acoustic shadowing, located in the stomach, pylorus, or small intestine. Gastrointestinal obstruction (GIO) was diagnosed based on ultrasound evidence of dilated bowel loops proximal to the bezoar, decreased or absent peristalsis, and/or retention of gastric contents. The diagnosis of GIO was further supported by corresponding clinical manifestations, including gastric retention (*n* = 22) confirmed by large-volume gastric aspirate or persistent vomiting, and intestinal obstruction (*n* = 14) presenting with complete cessation of flatus and defecation, abdominal distension, and air-fluid levels on plain abdominal radiography. The concordance between ultrasound findings and clinical presentation in all 36 GIO cases provides indirect validation of the ultrasound-based diagnosis. It should be noted that this study was not designed as a diagnostic accuracy study; confirmatory imaging (CT), endoscopy, surgery, or structured clinical follow-up were performed at the discretion of the treating clinician rather than as part of a standardized protocol. Therefore, the exact number and proportion of cases independently confirmed by CT, endoscopy, surgery, or clinical follow-up, stratified by GIO status and bezoar location, could not be determined from the retrospective dataset. Accordingly, cases in this manuscript are described as ultrasound-diagnosed phytobezoars rather than independently confirmed phytobezoars.

Ultrasound examination: all patients underwent abdominal ultrasound in the emergency department using Philips EPIQ 7 ultrasound system with a 3.5–5.0 MHz convex transducer. Patients were examined in the supine position. The stomach, pylorus, duodenum, and small intestine were systematically scanned. The location, number, and maximum diameter of bezoars were recorded. All examinations were performed by emergency sonographers with at least 3 years of experience in abdominal ultrasound.

Patients were categorized into two groups based on the presence of gastrointestinal obstruction: the obstruction group (GIO, *n* = 36) and the non-obstruction group (NGIO, *n* = 126). For analytical purposes, bezoar location was recorded using six original categories (gastric body, pylorus, small intestine, gastric body plus pylorus, gastric body plus small intestine, and pylorus plus small intestine) and subsequently dichotomized into “gastric body only” vs. “pylorus or small intestine involved” based on the primary site associated with obstruction risk. Comparative analyses were conducted between these groups. A binary logistic regression model was employed to identify factors associated with gastrointestinal obstruction in patients with bezoars.

Statistical analyses were performed using SPSS version 29.0 (IBM Corp., Armonk, NY, USA) and GraphPad Prism 10 (GraphPad Software, Inc., San Diego, CA, USA), and Python 3.12 (Python Software Foundation) with the SciPy library for sensitivity analyses. The independent *t*-test and Mann–Whitney *U*-test were utilized to compare groups with normally and non-normally distributed data, respectively. The Chi-square test was applied to analyze categorical data.

In the primary analysis, predictors of gastrointestinal obstruction were assessed using binary logistic regression analysis with a stepwise selection method. Bezoar location was excluded from the primary regression model due to complete separation of data (all GIO cases had bezoars in the pylorus or small intestine), which precluded valid estimation of regression coefficients using standard maximum likelihood estimation. The model fit was evaluated using the Hosmer–Lemeshow goodness-of-fit test. With 36 events and three predictor variables included in the final model, the events-per-variable (EPV) ratio was 12, meeting the minimum threshold recommended for logistic regression analysis.

To address the limitation of excluding bezoar location from the primary model, two pre-specified sensitivity analyses were performed. First, Firth penalized logistic regression was used to re-estimate the model with bezoar location included as a covariate. Firth's method adds a penalty term derived from the Jeffreys invariant prior to the likelihood function, which reduces the bias in maximum likelihood estimates and enables parameter estimation even in the presence of complete or quasi-complete separation (15). Second, a subgroup analysis was conducted restricted to patients with bezoars located in the pylorus or small intestine (*n* = 61), in which Firth logistic regression was used to examine whether sex, age, and bezoar diameter remained associated with GIO within this anatomically relevant subgroup. One patient in this subgroup had complete ultrasound imaging confirming the presence and location of a phytobezoar in the small intestine; however, the maximum diameter could not be reliably measured due to complete impaction within the intestinal lumen. This patient was included in all descriptive analyses (*n* = 162) but excluded from regression analyses requiring the diameter variable, yielding an analytical sample of 60.

A *P*-value of less than 0.05 was considered statistically significant.

## 3 Results

**Figure 1:**
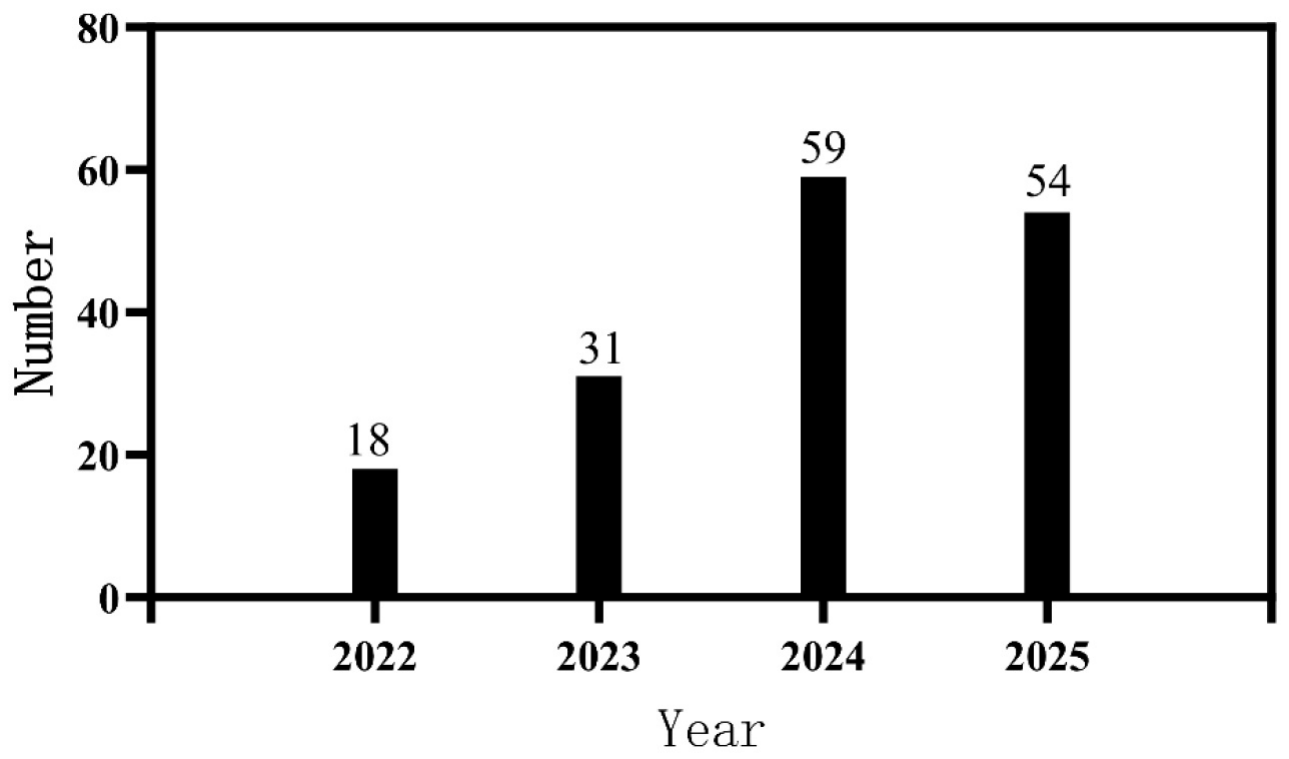
Number of patients by year.

**Figure 2:**
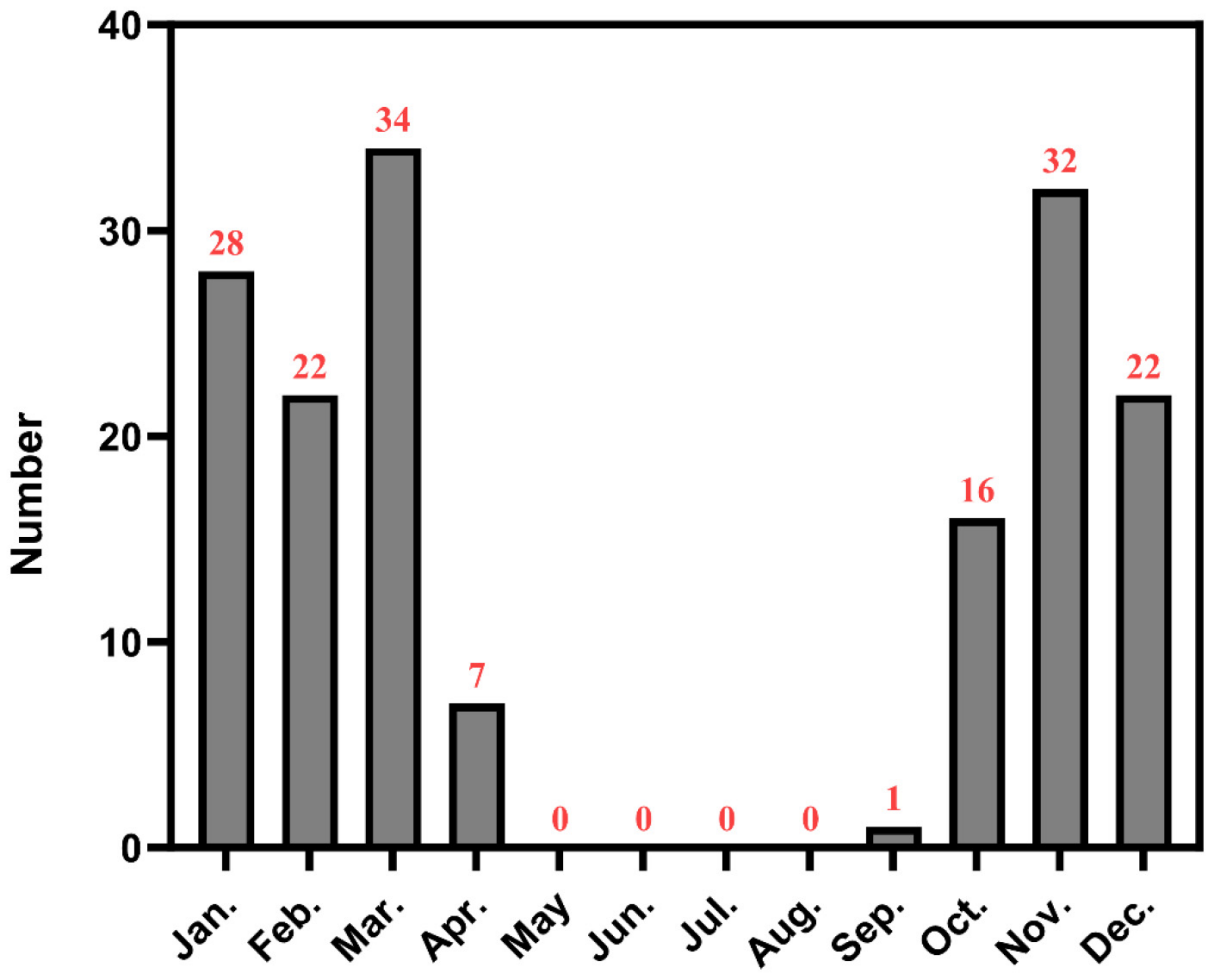
Number of patients by month.

The study encompassed 162 patients, all of whom reported a history of consuming hawthorn or persimmons and presented with abdominal pain. The age of the patients ranged from 21 to 81 years, with a median age of 64 years. The cohort comprised a higher proportion of female patients (*n* = 129, 79.6%) compared to male patients (*n* = 33, 20.4%). The incidence of bezoars has demonstrated an annual increase (Figure 1) and follows a distinct seasonal pattern (Figure 2), pre-dominantly from October to April of the following year, with a peak incidence in March (*n* = 34, 21.0%). A summary of patient characteristics is shown in Table 1.

**Table 1:** Characteristics of patients with phytobezoars.

Characteristics (*n* = 162)	*n*	%
Sex		
Female	129	79.6
Male	33	20.4
Year		
2022	18	11.1
2023	31	19.1
2024	59	36.5
2025	54	33.3
Month		
Jan	28	17.3
Feb	22	13.6
Mar	34	21.0
Apr	7	4.3
Sep	1	0.6
Oct	16	9.9
Nov	32	19.8
Dec	22	13.6

**Figure 3:**
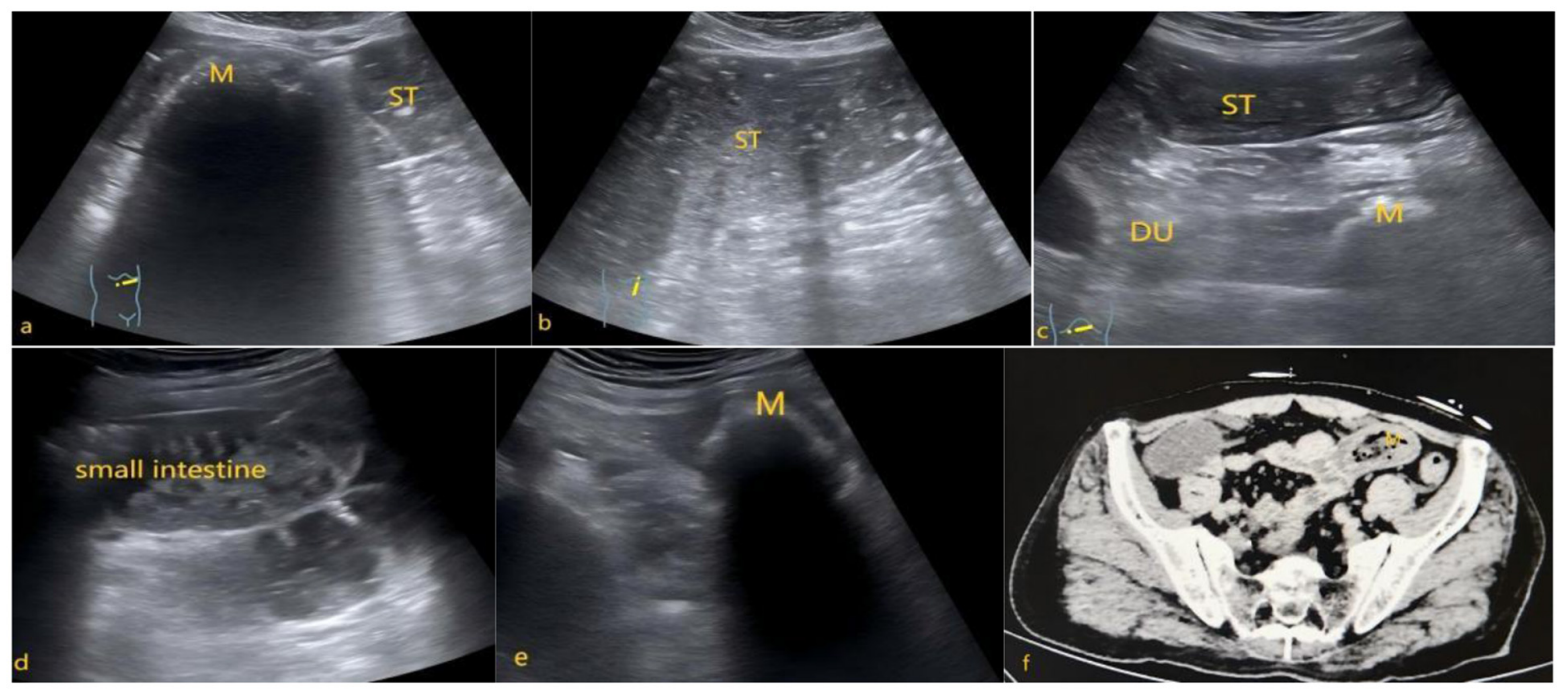
Ultrasonic characteristics of bezoars. Case 1: **(a)** Phytobezoar impacted in the pylorus; **(b)** Massive retention of gastric contents. Case 2: **(c)** Phytobezoar impacted in the horizontal part of the duodenum; **(d)** Later rechecking revealed jejunum obstruction; **(e)** A mass of bezoar was visible, followed by acoustic shadowing, impacted in the distal jejunum cavity; **(f)** CT also shows a gas-filled mass of bezoar in the left mid-abdominal jejunum. M, phytobezoar; ST, stomach; DU, duodenum.

Bezoars were located in the gastric body in 62.3% of cases, while 37.7% of cases involved migration to the pylorus or small intestine. Solitary bezoars were observed in 78.4% of cases, whereas multiple bezoars were present in 21.6% of cases. Additionally, 22.2% of cases experienced bezoar impaction, resulting in pyloric or small intestinal obstruction. The characteristics of bezoars are summarized in Table 2. Ultrasonic images of bezoars are shown in Figure 3.

**Table 2:** Ultrasonic characteristics of phytobezoars.

Characteristics (*n* = 162)	*n*	%
Location of phytobezoar		
Gastric body	101	62.3
Pylorus or small intestine	61	37.7
Number of phytobezoar		
Solitary	127	78.4
Multiple	35	21.6
Gastrointestinal obstruction		
Yes	36	22.2
No	126	77.8

**Figure 4:**
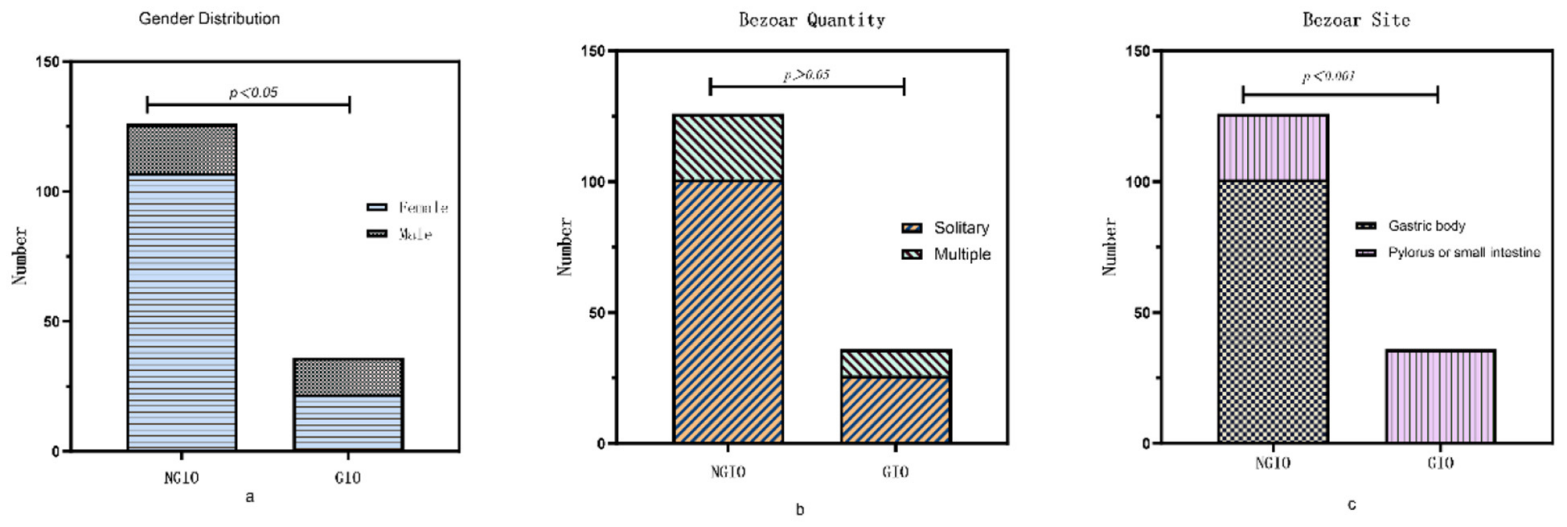
Between-group comparison of categorical variables. **(a)** Between-group comparison for sex distribution. **(b)** Between-group comparison for bezoar quantity. **(c)** Between-group comparison for bezoar location.

**Figure 5:**
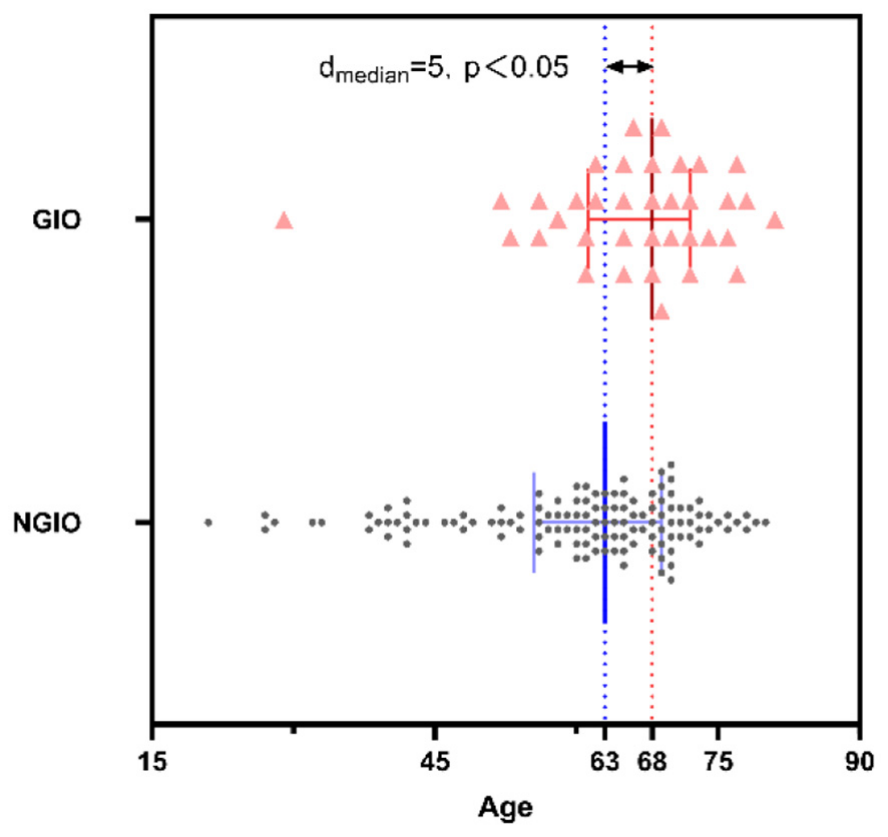
Between-group comparison of age.

**Figure 6:**
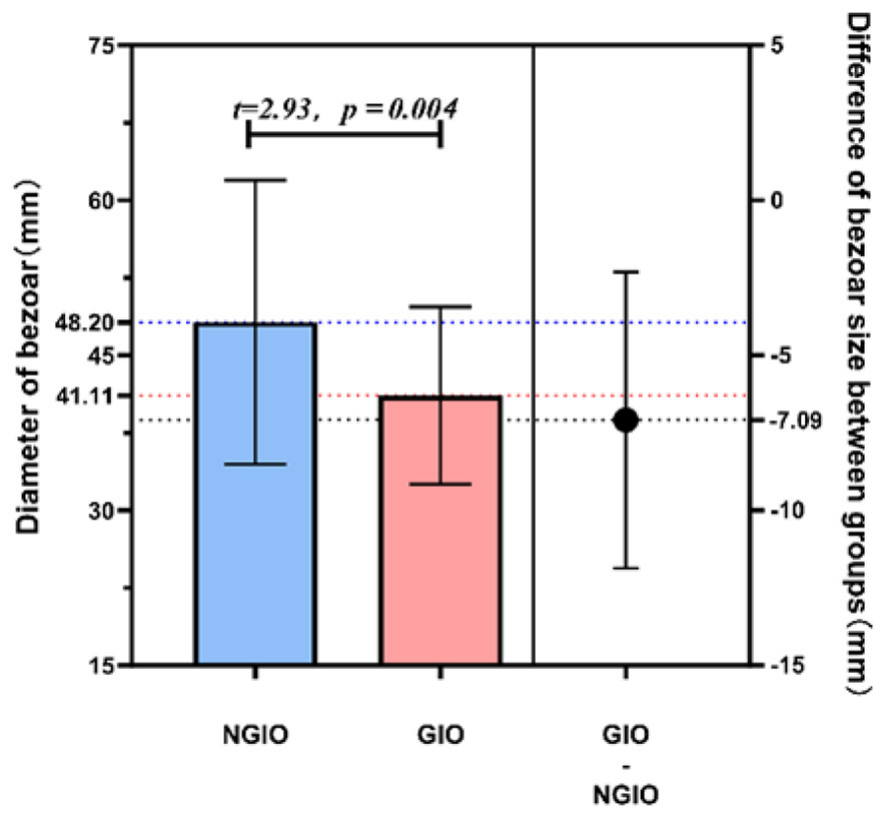
Between-group comparison of bezoar diameter.

A comparative analysis was performed to assess potential differences between the GIO group (*n* = 36) and the NGIO group (*n* = 126) concerning age, sex, bezoar diameter, quantity, and location. The analysis revealed significant differences between the two groups concerning sex distribution (*p* < 0.05) and the location of bezoars (*p* < 0.001), while no statistically significant difference was observed in the quantity of bezoars (*p* > 0.05, Table 3 and Figure 4). The median age of the GIO group was significantly higher than that of the NGIO group (68 vs. 63 years, *z*=-2.754, *p* = 0.006, Table 4 and Figure 5). The mean bezoar diameter in the GIO group was significantly smaller than in the NGIO group (41.11 ± 8.57 vs. 48.20 ± 13.75 mm, *t* = 2.93, *p* = 0.004, Table 4 and Figure 6).

**Table 3:** Between-group comparison of categorical variables.

Characteristics	NGIO group	GIO group	χ^2^	*p*-value
Sex				
Female	107	22	9.786	0.002
Male	19	14		
Bezoar quantity				
Solitary	101	26	1.041	0.308
Multiple	25	10		
Bezoar location				
Gastric body	101	0	97.637	< 0.001
Pylorus or small intestine	25	36		

GIO, gastrointestinal obstruction; NGIO, non-gastrointestinal obstruction.

**Table 4:** Between-group comparison for measurement data analysis.

Characteristics	NGIO group	GIO group	*t*/*z*	*p*-value
*N*	126	36		
Diameter of bezoar (mm)				
Mean ± SD	48.20 ± 13.75	41.11 ± 8.57	2.93	0.004
Min	15.00	26.00		
Max	81.00	57.00		
Age				
Median (IQR)	63 (25)	68 (8)	−2.754	0.006
Min	21	29		
Max	80	81		

GIO, gastrointestinal obstruction; NGIO, non-gastrointestinal obstruction; SD, standard deviation; IQR, interquartile range.

In the primary analysis, digestive tract obstruction was designated as the dependent variable, and a stepwise method was employed to identify independent variables. Sex, age, and bezoar diameter were selected as independent variables for binary logistic regression analysis. The Hosmer–Lemeshow goodness-of-fit test indicated that the model adequately fit the data (χ^2^ = 7.124, df = 8, *p* = 0.524).

**Figure 7:**
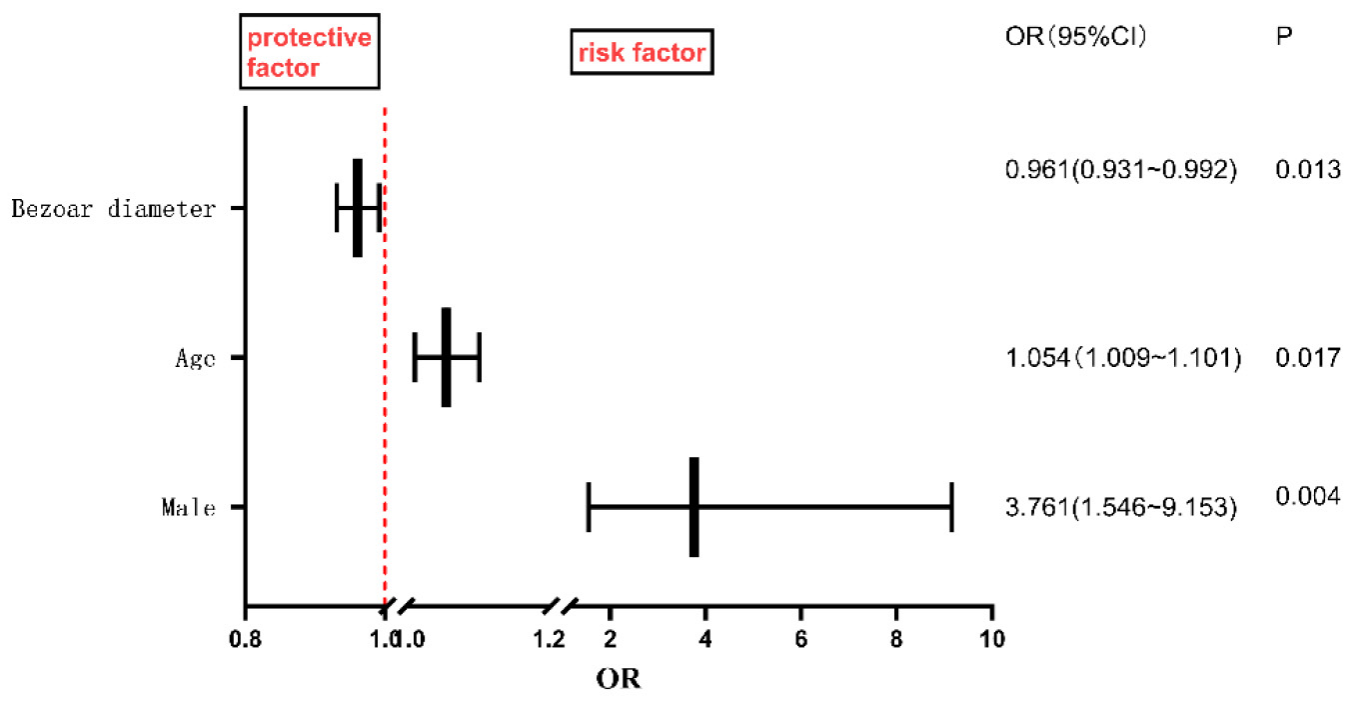
Forest plot of binary logistic regression analysis.

The probability of obstruction was calculated using the logistic regression equation: Logit (*P*) = −3.153 + 1.325 × sex (male = 1) + 0.053 × age – 0.040 × diameter of bezoar (Table 5 and Figure 7).

**Table 5:** Binary logistic regression analysis for gastrointestinal obstruction.

Parameter	β	Standard error	Wald	*p*-value	Odds ratio (95%C.I.)
Sex (male)	1.325	0.454	8.526	0.004	3.761 (1.546, 9.153)
Age	0.053	0.022	5.682	0.017	1.054 (1.009, 1.101)
Diameter of bezoar (mm)	−0.040	0.016	6.116	0.013	0.961 (0.931, 0.992)
Constant	−3.153	1.628	3.752	0.053	

Male sex (*OR* = 3.761, 95% CI: 1.546–9.153, *p* = 0.004), older age (*OR* = 1.054, 95% CI: 1.009–1.101, *p* = 0.017), and smaller bezoar diameter (*OR* = 0.961, 95% CI: 0.931–0.992, *p* = 0.013) were identified as significant predictors of gastrointestinal obstruction in the primary model (Table 5 and Figure 7). Specifically, male patients had approximately 3.8-fold higher odds of developing GIO compared to female patients. Each additional year of age was associated with a 5.4% increase in odds, whereas each 1 mm increase in bezoar diameter was associated with a 3.9% decrease in odds.

To address the complete separation of bezoar location in the primary model, two sensitivity analyses were performed.

First, Firth penalized logistic regression was conducted including bezoar location along with sex, age, and diameter as covariates (*n* = 161; one patient whose bezoar diameter could not be measured due to complete intestinal impaction was excluded). Bezoar location in the pylorus or small intestine was the dominant predictor of GIO (*OR* = 220.30, 95% CI: 15.02–3,231.46, *p* < 0.001). After adjustment for location, sex (*OR* = 2.78, 95% CI: 0.80–9.62, *p* = 0.106), age (*OR* = 1.04, 95% CI: 0.99–1.10, *p* = 0.089), and bezoar diameter (*OR* = 1.00, 95% CI: 0.95–1.05, *p* = 0.931) were no longer statistically significant (Table 6).

**Table 6:** Sensitivity analyses: firth penalized logistic regression.

Variable	Model 1: full sample with location (*n* = 161)		*p*	Model 2: pylorus/SI subgroup (*n* = 60)		*p*
	β	OR (95% CI)		β	OR (95% CI)	
Sex (male)	1.023	2.78 (0.80–9.62)	0.106	1.013	2.75 (0.77–9.82)	0.118
Age (years)	0.043	1.04 (0.99–1.10)	0.089	0.043	1.04 (0.99–1.10)	0.095
Diameter (mm)	−0.002	1.00 (0.95–1.05)	0.931	−0.002	1.00 (0.95–1.05)	0.940
Location (pylorus/SI)	5.395	220.30 (15.02–3,231.46)	< 0.001	—	—	—

Model 1: firth penalized logistic regression including all four variables in the full sample.

Model 2: firth penalized logistic regression restricted to patients with bezoars in the pylorus or small intestine (location variable excluded as all patients shared the same category).

One patient with missing diameter data was excluded from each model.

Second, a subgroup analysis was restricted to patients with bezoars located in the pylorus or small intestine (*n* = 61). Within this subgroup, univariate comparisons showed no statistically significant differences between GIO (*n* = 36) and NGIO (*n* = 25) patients in sex (male: 38.9% vs. 16.0%, *p* = 0.101), age (median: 68 vs. 63 years, *p* = 0.086), or bezoar diameter (mean: 41.26 ± 8.65 vs. 42.32 ± 12.52 mm, *p* = 0.699). Firth logistic regression within this subgroup (*n* = 60; one patient excluded for missing diameter) confirmed that none of the three variables reached statistical significance: sex (*OR* = 2.75, 95% CI: 0.77–9.82, *p* = 0.118), age (*OR* = 1.04, 95% CI: 0.99–1.10, *p* = 0.095), and diameter (*OR* = 1.00, 95% CI: 0.95–1.05, *p* = 0.940; Table 6).

These findings indicate that bezoar location was the overwhelmingly dominant predictor of GIO. The associations of sex, age, and diameter observed in the primary model were substantially attenuated after accounting for location, suggesting that these associations may have been partly confounded by the strong relationship between bezoar location and both patient characteristics and obstruction risk.

## 4 Discussion

### 4.1 Epidemiological characteristics and seasonal patterns

Bezoars refer to lumps formed by the accumulation of undigested substances within the gastrointestinal tract, with phytobezoars and trichobezoars being the most prevalent types. The earliest documented case of bezoars dates back to 1779, while the first recorded instance of phytobezoars was in 1854 (16). Phytobezoars are the most common type of bezoar, and they have been reported to account for the majority of all bezoar cases worldwide (2, 3, 17). The incidence of phytobezoars varies across different regions and populations. The hospital associated with this study is situated in North China, where foods such as hawthorn, persimmons, and black jujubes, which can precipitate phytobezoars, are plentiful during the fall and winter. This seasonal abundance coincides with the peak incidence period of phytobezoars observed in this study. The annual increase in the number of cases may be attributed to several factors. First, the implementation of systematic emergency gastrointestinal ultrasound protocols at our institution since 2019 has improved detection rates. Second, modifications in medical insurance policies covering emergency visits may have lowered the threshold for seeking medical attention. Third, growing public awareness of the association between persimmon or hawthorn consumption and gastrointestinal complications has likely encouraged more patients to present for evaluation. Similar trends of increasing detection have been reported in other regions where awareness and diagnostic capabilities have improved (14, 18).

### 4.2 Age and sex distribution

The majority of patients were elderly, with a median or mean age of approximately 60 years, aligning with previous studies (19). Several factors may account for this trend. Firstly, elderly individuals often experience physiological declines in chewing ability and gastrointestinal digestive function, resulting in prolonged gastric emptying times and an increased susceptibility to bezoar formation. Secondly, risk factors for bezoar formation, such as diabetes, hypertension, and a history of digestive tract diseases, are more prevalent in the elderly population. Thirdly, in China, hawthorn is traditionally believed to possess properties that strengthen the stomach, promote digestion, lower blood pressure, and enhance metabolism. In this study, the majority of elderly individuals consumed raw hawthorn for these purported benefits, which contributed to the development of phytobezoars.

In terms of gender distribution, earlier research suggested a higher prevalence of phytobezoars in males compared to females (20). However, the present study reveals a significantly greater incidence of phytobezoars in females, aligning with findings from some recent clinical investigations (14, 21). This discrepancy may be attributed to a dietary preference among females for foods known to contribute to phytobezoar formation, such as hawthorn or persimmons.

Notably, although females constituted the majority of phytobezoar patients in this cohort, male sex was associated with a higher odds of gastrointestinal obstruction in the primary analysis. We hypothesize that this observation may partly relate to gender-related differences in gastric motility. Studies have demonstrated that men have significantly faster gastric emptying rates compared to women, both for solid and liquid meals (22–24). Faster gastric emptying may theoretically facilitate the passage of smaller, partially formed bezoar fragments through the pylorus into the narrower small intestinal lumen, thereby increasing the likelihood of impaction and obstruction. In contrast, the slower gastric emptying in women may allow bezoars to be retained in the gastric body for a longer period, where they continue to grow but are less likely to migrate distally. However, it should be noted that gastric motility and dietary patterns were not directly measured in the present study, and these mechanistic explanations remain speculative. Furthermore, the sensitivity analysis restricted to patients with pyloric or small-intestinal bezoars showed that the association between male sex and GIO was attenuated and no longer statistically significant (*OR* = 2.75, *p* = 0.118), suggesting that this association may be partly confounded by bezoar location rather than representing a truly independent effect.

### 4.3 Role of emergency ultrasound in diagnosis

Timely diagnosis and intervention are crucial, as delays can result in severe complications, including injury to the digestive tract, bleeding, or even obstruction. Given that gastrointestinal obstruction is the most common and serious complication of bezoars (5), early detection of high-risk patients is of particular clinical importance. Consequently, early detection and management of bezoars to prevent gastrointestinal obstruction are of significant importance. Emergency ultrasound is instrumental in the early non-invasive screening of bezoars, facilitating the assessment of their size, quantity, and location, as well as the evaluation of potential gastrointestinal obstruction. Moreover, unlike CT, ultrasound can be performed at the bedside repeatedly without radiation exposure, which may be advantageous for follow-up assessment during conservative treatment (10, 11). However, dynamic monitoring during treatment was not evaluated in the present study. A previous study reported that ultrasound could detect bezoars in 88% of cases, with characteristic features including high echogenicity and posterior acoustic shadowing (10, 12). However, it should be acknowledged that the present study was not designed to evaluate the diagnostic accuracy of ultrasound for phytobezoars or GIO. All diagnoses were based on emergency ultrasound without systematic comparison against CT or endoscopic findings, and therefore the sensitivity and specificity of ultrasound in this clinical context could not be determined. Despite this limitation, the clinical utility of emergency ultrasound in this setting lies in its ability to rapidly identify bezoar location, which our data indicate is the most important determinant of obstruction risk.

Taken together, our findings suggest that the primary clinical value of emergency ultrasound in patients with ultrasound-diagnosed phytobezoars lies not in profiling patient-level risk characteristics, but in the rapid bedside identification of bezoar location. Since location was the overwhelmingly dominant predictor of GIO-and the only variable that retained significance across all analytical approaches-the ability of ultrasound to instantly determine whether a bezoar resides in the gastric body or has migrated to the pylorus or small intestine represents the most actionable clinical information for emergency physicians.

### 4.4 Risk factors for gastrointestinal obstruction

Utilizing binary logistic regression analysis, the primary model in this study identified male sex and age as factors associated with gastrointestinal obstruction among patients with ultrasound-diagnosed phytobezoars. The likelihood of gastrointestinal obstruction is greater in males compared to females, and this risk escalates with advancing age, which contrasts with findings from a prior study. A clinical investigation into gastrointestinal obstruction caused by bezoars in the elderly population reported no statistically significant differences in gender distribution, bezoar size, or obstruction location between the elderly group (aged ≥65 years) and the younger group (aged < 65 years). However, the elderly group exhibited a higher incidence of complications and experienced longer hospital stays (13).

Concurrently, bezoar diameter was identified as a protective factor against gastrointestinal obstruction. This finding can be explained by a straightforward anatomical mechanism: larger bezoars are physically unable to pass through the pylorus (approximately 12–15 mm in diameter) and therefore tend to remain in the gastric body, where they cause symptoms but do not obstruct the intestinal lumen (25, 26). Conversely, smaller bezoars or bezoar fragments are more likely to migrate into the narrower pyloric canal or small intestine, where impaction and obstruction can occur. This observation is corroborated by previous studies, which indicate that bezoars located in the stomach are generally larger than those found in the small intestine (10). Additionally, earlier case studies have associated bezoar size with factors such as dietary intake, gastric acid pH, and a history of gastric surgery (25). It has also been documented that in certain cases, large bezoars have migrated into the small intestine following treatment, resulting in gastrointestinal obstruction and necessitating laparoscopic surgery (27). This underscores the importance of vigilant monitoring of patients with bezoars during treatment.

It is noteworthy that bezoar location exhibited complete separation in our data: all 36 patients with GIO had bezoars located in the pylorus or small intestine, whereas all 101 patients with bezoars in the gastric body were free of obstruction. Although this distribution precluded inclusion of location in the logistic regression model, it represents the most clinically significant finding of this study. The Firth penalized logistic regression, which enabled inclusion of location despite complete separation, confirmed that location was the overwhelmingly dominant predictor of GIO (*OR* = 220.30, *p* < 0.001), while sex, age, and diameter were no longer statistically significant after adjustment for location. Consistent with this, the subgroup analysis restricted to patients with pyloric *or* small-intestinal bezoars showed that none of these three variables retained a significant association with GIO. These results suggest that the associations observed in the primary model may have been partly confounded by bezoar location. Specifically, bezoars confined to the gastric body were larger (mean 49.65 mm, *n* = 101) than those in the pylorus or small intestine (mean 42.32 mm in NGIO and 41.26 mm in GIO within this subgroup), which likely drove the apparent protective effect of larger diameter. Similarly, the higher proportion of female patients with gastric body bezoars may have contributed to the observed male pre-dominance in the GIO group.

Nonetheless, it should be noted that sex and age showed borderline trends toward significance in the sensitivity analyses (*p* = 0.106–0.118 and *p* = 0.089–0.095, respectively), and the limited sample size within the subgroup (*n* = 61) may have resulted in insufficient statistical power to detect modest effects. Future studies with larger sample sizes are needed to clarify whether these variables contribute independently to obstruction risk beyond the effect of location.

Additionally, several well-established risk factors for bezoar formation and obstruction, including history of gastric surgery, diabetes mellitus, gastroparesis, and use of anticholinergic medications (3, 28), could not be included in the analysis due to incomplete documentation in the emergency medical records. This represents an important limitation and means that residual confounding cannot be excluded in the current model.

In clinical practice, the detection of a bezoar in the pylorus or small intestine on emergency ultrasound should prompt immediate clinical vigilance for potential obstruction, regardless of other patient characteristics.

### 4.5 Strengths and limitations

This study examines the demographic characteristics and ultrasonic findings of patients with phytobezoars to explore factors associated with gastrointestinal obstruction. The goal is to highlight clinically relevant ultrasound findings associated with GIO and to support early clinical recognition of patients at risk of obstruction. The novelty of this study lies in its focus on ultrasonic imaging characteristics of bezoars, a perspective not extensively explored in previous research, which has pre-dominantly concentrated on medical history, treatment strategies, or prognosis. However, this study has several limitations. First, it is a single-center retrospective study with a relatively small GIO group (*n* = 36). Although the events-per-variable ratio of 12 meets the conventional minimum threshold for logistic regression (29), the limited sample size may have reduced statistical power. This was particularly evident in the subgroup sensitivity analysis (*n* = 61), where sex and age showed borderline trends (*p* = 0.089–0.118) that might reach significance in a larger cohort.

Second, both the exposure (phytobezoar diagnosis) and the outcome (GIO) were defined by ultrasound criteria within the same imaging framework. Although the diagnosis of GIO was supported by corresponding clinical manifestations documented in the medical records, this study was not designed as a diagnostic accuracy study, and ultrasound findings were not systematically validated against CT, endoscopy, surgery, or structured clinical follow-up. The exact number and proportion of cases confirmed by these independent reference methods, stratified by GIO status and bezoar location, could not be determined because verification was not standardized and was not consistently documented in the analyzable dataset. The possibility of misclassification therefore cannot be excluded, particularly for gastric lesions. Previous studies have reported that ultrasound can detect bezoars in approximately 88% of cases with characteristic features (10), which supports the overall reliability of the approach, but prospective studies incorporating cross-modal validation are needed.

Third, this study identified cases by comprehensive keyword search of the emergency ultrasound database, which archives all examinations performed in the emergency department. Although the total number of emergency abdominal ultrasound examinations during the study period was not separately tallied in the database system, the search-based approach ensures that all ultrasound-diagnosed phytobezoar cases were captured. The annual caseload increased steadily from 18 cases in 2022 to 59 cases in 2024, which is consistent with the institution's expanding emergency ultrasound program and growing clinical awareness rather than reflecting an unusually high detection rate.

Fourth, the inclusion criterion requiring a self-reported history of consuming hawthorn, persimmons, or black jujubes introduces potential recall bias. However, given the strong and well-recognized association between these specific foods and phytobezoar formation, and the typically short interval between consumption and symptom onset, patients are generally able to recall their recent dietary intake with reasonable accuracy.

Fifth, several well-established risk factors for bezoar formation and obstruction, including history of gastric surgery, diabetes mellitus, gastroparesis, and use of anticholinergic medications (3, 28), could not be included in the regression model due to incomplete documentation in the emergency medical records. The emergency clinical setting prioritizes acute assessment, and chronic medical history is often incompletely recorded. This limitation means that residual confounding cannot be excluded, and the observed associations should be interpreted with caution in the context of this study.

Sixth, bezoar location could not be included in the primary regression analysis due to complete separation of data. Although Firth penalized regression and subgroup sensitivity analyses were performed to address this limitation, these supplementary analyses demonstrated that the associations observed in the primary model were substantially attenuated after accounting for location, indicating that the primary model results should be interpreted in light of the dominant effect of bezoar location.

Seventh, the analysis was restricted to ultrasound data obtained at initial presentation and did not incorporate dynamic follow-up observations or treatment outcomes. Therefore, claims regarding the value of ultrasound for dynamic monitoring remain hypothetical and require prospective validation.

Eighth, inter-observer reliability assessment among sonographers was not performed. Although all examinations were conducted by emergency sonographers with at least 3 years of experience in abdominal ultrasound, the absence of formal intra-class correlation coefficient evaluation means that measurement variability in bezoar diameter estimation cannot be quantified. Future studies should incorporate inter-observer reliability assessment to strengthen the validity of diameter-based analyses.

### 4.6 Future directions

Future research should consider multi-center studies with larger sample sizes to validate these findings. Prospective studies incorporating dynamic ultrasound monitoring throughout the treatment course would provide valuable insights into bezoar progression and treatment response. Additionally, future studies should evaluate whether combining ultrasound parameters with clinical factors can improve the assessment of obstruction risk in patients with phytobezoars.

## 5 Conclusion

The occurrence of phytobezoars demonstrates a distinct seasonal pattern, aligning with the periods of consumption of foods known to induce phytobezoars. Among patients with ultrasound-diagnosed phytobezoars, bezoar location was the most important determinant of gastrointestinal obstruction: all obstruction cases occurred in patients with bezoars in the pylorus or small intestine, whereas no gastric body bezoars were associated with obstruction. Although the primary regression model identified male sex, older age, and smaller bezoar diameter as factors associated with obstruction, sensitivity analyses indicated that these associations were substantially attenuated after accounting for bezoar location and may have been partly confounded by it. Emergency ultrasound provides a rapid, non-invasive means of identifying bezoar location at the bedside, which may assist clinicians in the early recognition of patients at higher risk for obstruction. Prospective multi-center studies with larger sample sizes are needed to validate these findings and to evaluate the role of dynamic ultrasound monitoring in guiding treatment decisions.

## Data Availability

The raw data supporting the conclusions of this article will be made available by the authors, without undue reservation.
